# Efficacy of Continuous Positive Airway Pressure Therapy in a Patient With Saber-Sheath Trachea on Dilated Central and Peripheral Airways: A Case Report

**DOI:** 10.7759/cureus.74266

**Published:** 2024-11-22

**Authors:** Mitsuhiro Yamamoto, Tomoyuki Ikeuchi, Hirokazu Touge, Katsuyuki Tomita, Akira Yamasaki

**Affiliations:** 1 Department of Respiratory Medicine, National Hospital Organization (NHO) Yonago Medical Center, Yonago, JPN; 2 Department of Multidisciplinary Internal Medicine, Division of Respiratory Medicine and Rheumatology, School of Medicine, Faculty of Medicine, Tottori University, Tottori, JPN

**Keywords:** airway trapping, chronic bronchitis, continuous positive airway pressure (cpap), copd: chronic obstructive pulmonary disease, saber-sheath trachea

## Abstract

A saber-sheath trachea is a type of tracheal deformity characterized by coronal narrowing and sagittal widening of the intrathoracic trachea. In this case report, we describe a 76-year-old man with a history of chronic obstructive pulmonary disease (COPD) and repeated episodes of type 2 respiratory failure that responded poorly to inhaled long-acting β2 agonists, long-acting muscarinic antagonists, and corticosteroids. The patient was admitted to our hospital due to a COPD exacerbation. Chest computed tomography (CT) demonstrated marked coronal shortening and narrowing, sagittal widening of the intrathoracic trachea, and bronchiolitis. The tracheal index (TI) was less than two-thirds. Continuous positive airway pressure (CPAP) therapy resolved the type 2 respiratory failure, effectively improving the TI in the dilated coronal trachea. Considering the percentage of low-attenuation areas, airway trapping was observed in CT images. This case report demonstrated that CPAP therapy may improve the narrowing of the central and peripheral airways.

## Introduction

The saber-sheath trachea is a rare clinical condition. It is characterized by coronal narrowing (less than two-thirds of the sagittal diameter) of the intrathoracic trachea. Changes in the intrathoracic tracheal dimensions and shape are also commonly seen in airway obstruction, such as chronic bronchitis (a form of chronic obstructive pulmonary disease (COPD)), emphysema, hyperinflation, and bronchiolitis obliterans syndrome after lung transplantation [1,2]. The saber-sheath trachea is most frequently associated with chronic bronchitis and is common in male smokers over the age of 50 years [3]. Moreover, it is often used as a diagnostic parameter for chronic bronchitis, with a specificity of 92.9％ and a sensitivity of 39.1% [4]. Furthermore, the saber-sheath trachea appears to be related to the functional severity of central airway obstruction and peripheral air trapping [3]. Factors contributing to the formation of the saber-sheath trachea include repetitive cartilaginous injury due to excessive coughing, increased intrathoracic pressure, and deformability associated with peripheral airway diseases such as bronchiolitis. Although the relationship between the saber-sheath trachea and chronic bronchitis has long been recognized, the detailed pathophysiological mechanisms remain unknown. In this case report, we describe a 76-year-old man with a saber-sheath trachea and repeated episodes of type 2 respiratory failure, central tracheal deformity, and peripheral airway tapping, who was successfully managed with continuous positive airway pressure (CPAP) therapy.

## Case presentation

**Figure 1:**
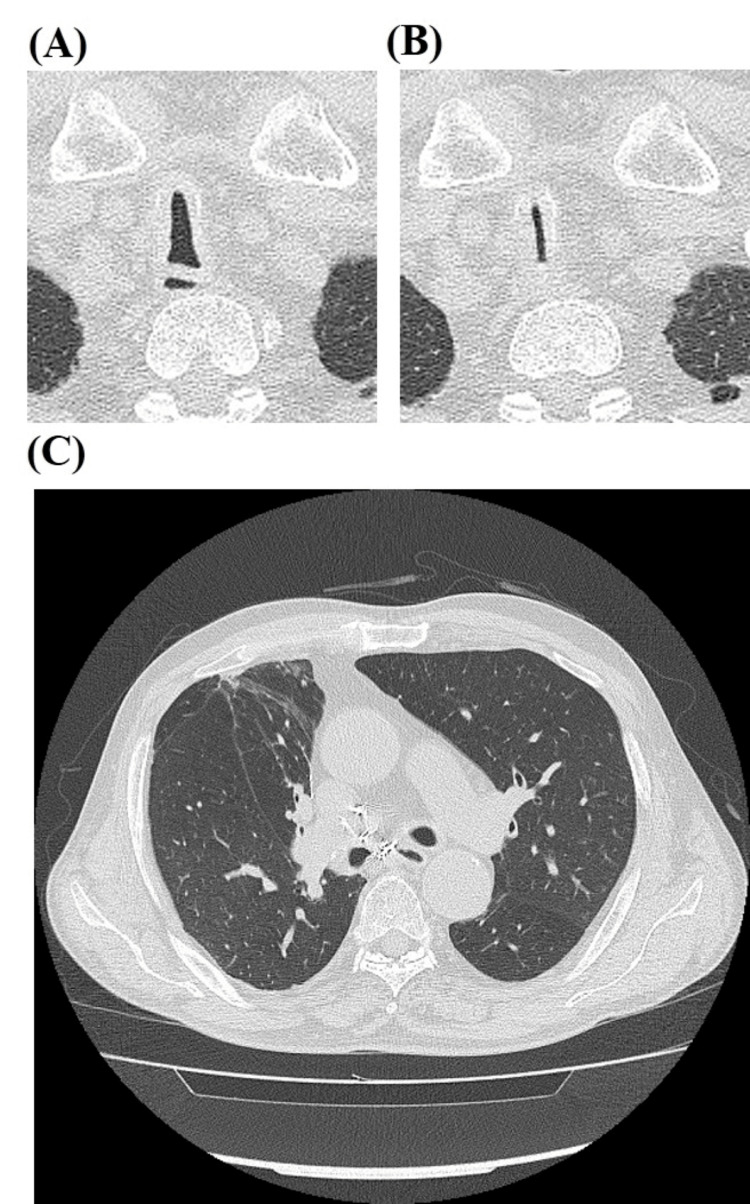
Chest computed tomography (CT) in the inspiration (A) and expiration (B) phases showing the saber-sheath trachea 1 cm above the aortic arch. (C) High-resolution CT demonstrating centrilobular nodules and bronchiolar wall thickening in the peripheral airways.

**Figure 2:**
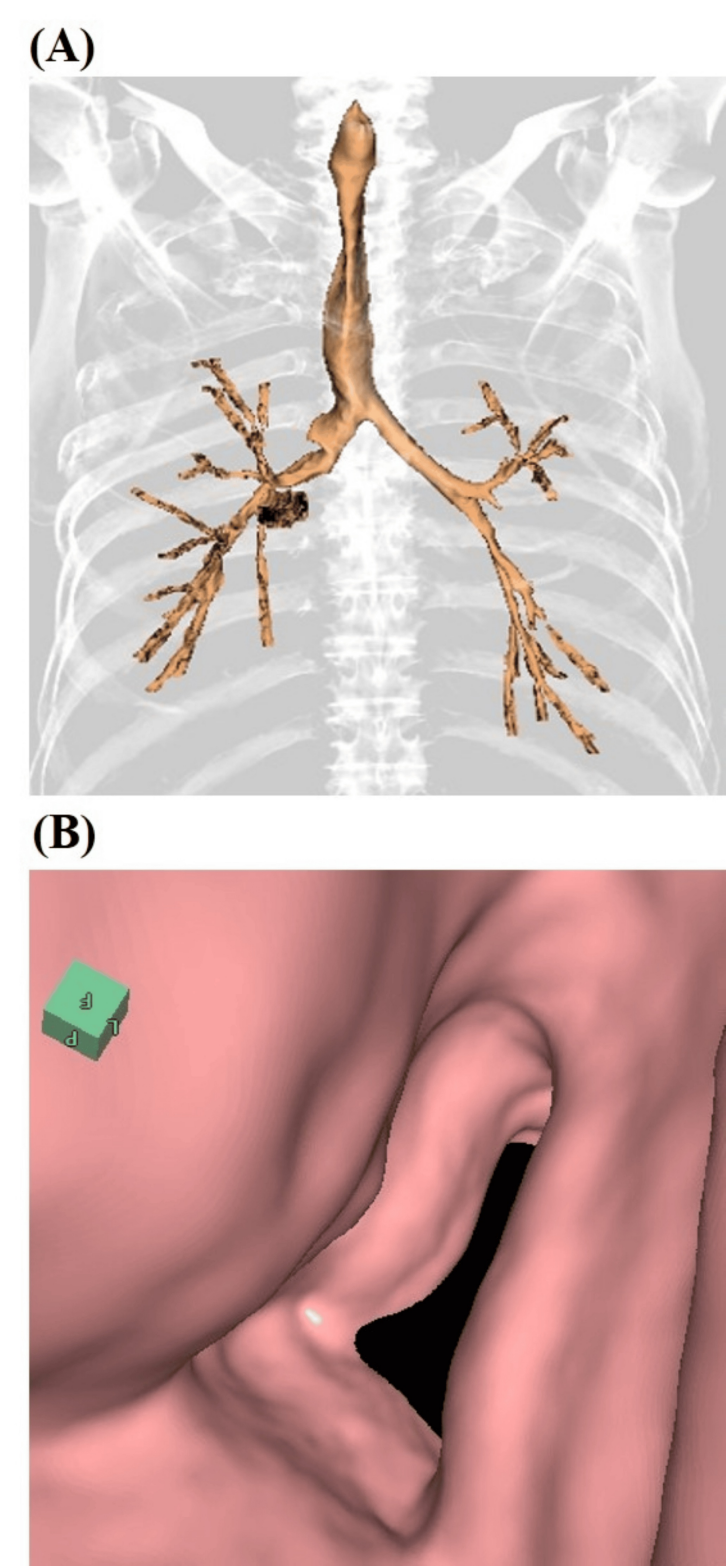
Coronal (A) and virtual (B) bronchoscopy images of the saber-sheath trachea on a dimensional chest computed tomography reconstruction image.

A 76-year-old man with a 30-pack-year smoking history and severe COPD (Global Initiative for Chronic Obstructive Lung Disease (GOLD) stage 3) presented to the emergency department (ED) of our hospital with worsening dyspnea for more than five days. His regular medications included triple-inhalant therapy (long-acting β2 agonists + long-acting muscarinic antagonists + corticosteroids). The patient had experienced repeated episodes of type 2 respiratory failure that did not respond to the triple-inhalant therapy. Upon observation in the ED, the patient was afebrile. His vital signs were as follows: pulse rate of 96 beats per minute, blood pressure of 178/104 mmHg, respiratory rate of 32 breaths per minute, and oxygen saturation of 96% on a nasal cannula at 1 L/min. Auscultation revealed expiratory airway narrowing sounds in the neck and diminished breath sounds in the chest. Laboratory tests revealed a strong inflammatory response, with a C-reactive protein (CRP) level of 21.3 mg/dL (reference range, <0.5 mg/dL). Arterial blood gas analysis indicated marked respiratory acidosis with metabolic alkalosis compensation (pH 7.1, PaCO_2_ 94.9 torr, PaO_2_ 101.4 torr, and H_2_CO_3_⁻ 29.0 mmol/L) while the patient was receiving 1 L/min of nasal cannula oxygen therapy. Chest computed tomography (CT) showed marked coronal shortening and narrowing, as well as sagittal widening of the intrathoracic trachea. The tracheal index (TI) was 0.44 during the inspiration (Figure 1A) and 0.11 during the expiration (Figure 1B). High-resolution CT (HRCT; Figure 1C) revealed centrilobular nodules and bronchiolar wall thickening in the peripheral airways, suggestive of infectious bronchiolitis. Virtual bronchoscopy showed progressive narrowing of the trachea to 4 cm in length from the thoracic outlet (Figure 2A and Figure 2B).

The patient was admitted to our hospital with a diagnosis of infectious bronchiolitis and a saber-sheath trachea. Antimicrobial therapy with intravenous sulbactam/cefoperazone (9 g/day) was initiated. CPAP therapy using noninvasive ventilation was selected to act as a pneumatic stent to decrease pulmonary resistance and improve expiratory airflow obstruction [5]. Our patient was treated with a positive pressure of 8 cm H_2_O on the first day of hospitalization. On day 3 of admission, the patient was escalated to invasive ventilation to assist with his breathing and secure the airway, as he was producing a large amount of sputum and could no longer maintain oxygen saturation (SpO_2_) above 90%, despite the positive pressure of 8 cm H_2_O on CPAP therapy. After intubation, invasive ventilation was initially set with a positive end-expiratory pressure (PEEP) of 10 cm H_2_O. Subsequently, pulmonary oxygenation gradually improved. On day 8 of admission, PEEP was reduced to 6 cm H_2_O. Chest radiography showed no lung abnormalities, while bronchoscopy revealed an accumulation of secretions in the central airways. On day 9 of admission, the patient was extubated and managed with a positive pressure of 4 cm H_2_O using CPAP with noninvasive ventilation.

**Figure 3:**
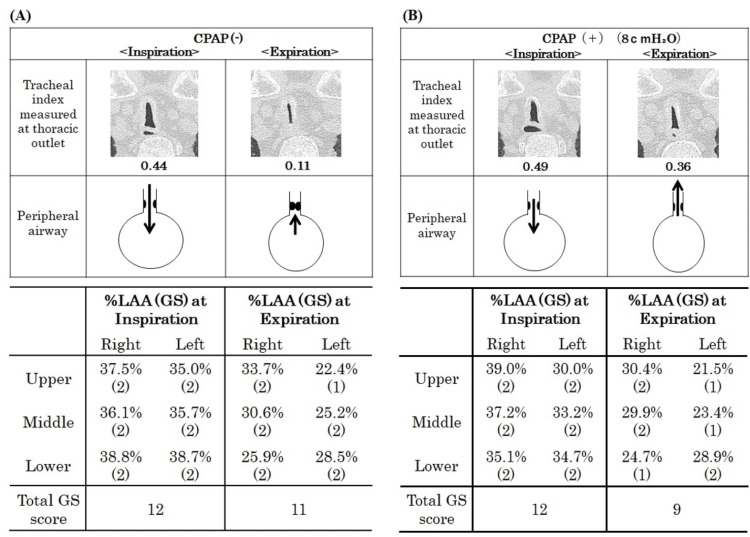
Assessment of the tracheal index (TI) of the saber-sheath trachea at the thoracic outlet, % low attenuation area (%LAA), and Goddard score (GS) in the lung field without CPAP (A) and with CPAP 8 cm H₂O (B). The GS is calculated as the proportion of the LAA for each area. Three sections make up the GS: the upper (aortic arch level), middle (carina level), and lower (upper end of the diaphragm level) [6]. The lung field area with attenuation values less than −950 Hounsfield units (HU) of thresholds is considered as the LAA. Each of these six sections uses a 4-point scale to assess the severity of the LAA: 1 (LAA occupying <25%), 2 (25% to <50%), 3 (50% to <75%), and 4 (≥75 %). The total GS are calculated by summing the scores for the six areas. These scores are automatically assessed using the SYNAPSE VINCENT system (Fujifilm Medical Service Solution Co., Ltd., Tokyo, Japan).

Following respiratory stabilization on day 15 of admission, the patient’s spirometry results showed a forced expiratory volume in 1 s (FEV_1.0_) of 1.05 L (%predicted FEV_1.0_, 47.5%), forced vital capacity (FVC) of 2.21 L (%FVC 65.8%), and diffusing capacity for carbon monoxide/alveolar ventilation (DLco/VA) of 2.99 mL/min/mmHg/L (%DLco/VA 70.7％). A single-breath nitrogen test demonstrated an N_2_ slope of 4.36 and a closing volume/vital capacity of 35.39%. An air trapping index of 6.4% indicated severe obstruction with hyperinflation, ventilation-perfusion mismatch, and low gas transfer. We also assessed the central airway obstruction using TI and peripheral airway trapping using a low-attenuation area (LAA) with a threshold of -950 Hounsfield units (HU) in HRCT to assess the effectiveness of CPAP. The TI was defined as the coronal/sagittal diameter measured 1 cm above the aortic arch. Figure 3A shows that the patient’s TI at the level of the thoracic outlet was smaller than at 1 cm above the aortic arch. The TI at the thoracic outlet level was considered more severe in this case. Therefore, we compared the TI before and after CPAP use at the thoracic outlet level. The use of CPAP dilated the central airways, resulting in a change in TI from 0.44 to 0.49 during inspiration and from 0.11 to 0.36 during expiration (Figure 3B). When the severity of the LAA was assessed using the total Goddard score (GS) [6] in the lung field during inspiration and expiration, the results indicated that CPAP reduced the severity of the LAA during expiration (Figure 3).

At the time of discharge, CPAP was no longer required. However, the patient experienced recurrent wheezing and dyspnea after CPAP withdrawal. As a result, the patient returned to the hospital, and home CPAP (at a positive pressure of 8 cm H_2_O) was reinitiated.

## Discussion

The saber-sheath trachea was first described by Greene and Lechner in 1975 and is characterized by a reduction in the coronal diameter and elongation of the sagittal diameter of the intrathoracic trachea [7]. This rare condition is primarily associated with chronic bronchitis. Lechner investigated a group of 90 men and women with pulmonary function indicative of severe COPD and found that patients with chronic cough and chronic bronchitis tend to have intrathoracic coronal narrowing and sagittal widening, which are consistent with the saber-sheath deformity [8].

In our case, infectious bronchiolitis may have worsened the saber-sheath trachea against the background of central airway collapse. Additionally, airway secretion increases with chronic lower airway inflammation, triggering airflow obstruction and progressing to air trapping, which in turn increases the levels of endogenous PEEP, also known as auto-PEEP. The central airway collapse and air trapping were indicative of a worsening saber-sheath trachea. We used TI as a measure of central airway collapse and the reduction rate of the %LAA between inspiration and expiration as a measure of air trapping. CPAP treatment improved the TI and %LAA. As shown in Figure 3, CPAP reduced the total GS during expiration. This demonstrates that CPAP treatment was effective against the auto-PEEP associated with air trapping. An increase in auto-PEEP is a burden on the inspiratory muscles and causes respiratory muscle fatigue, resulting in decreased ventilation. Adding PEEP to match the auto-PEEP reduces the respiratory workload and increases the ventilation volume [9,10]. PEEP can also be used as an adjunct therapy to facilitate secretion drainage [11].

The TI (Figure 3), measured at the thoracic outlet level, tended to improve with CPAP during both inspiration and expiration. We demonstrated that CPAP prevented central airway collapse in the saber-sheath trachea. PEEP is effective as a conservative therapy in cases of stenosis associated with central airway softening, where it reduces airway retention, while CPAP helps prevent airway collapse, particularly during expiration, and maintains effective ventilation [12,13].

We did not use bi-level positive airway pressure (BiPAP) but instead used CPAP to improve symptoms and acidosis. When BiPAP is used conservatively in patients with central airway softening, the focus is often on the potential for significant respiratory muscle fatigue, where CPAP alone may not be sufficient to improve airway collapse and provide adequate ventilation. With its two-phase pressures, BiPAP can significantly reduce respiratory muscle workload by providing ventilatory support. The main pathophysiology in our patient was presumed to be sputum retention due to infectious bronchiolitis, rather than respiratory muscle fatigue. Moreover, metabolic compensation was adequate. Therefore, respiratory management using BiPAP was not necessary, as the patient's respiratory status improved with CPAP alone, in combination with antimicrobial therapy.

## Conclusions

Abnormal airway collapse of the saber-sheath trachea is associated with functionally severe airway obstruction. The saber-sheath trachea should be considered in patients with chronic bronchitis who experience clinical deterioration. While the saber-sheath trachea is often associated with chronic bronchitis, its exact pathogenesis remains unknown. In our case, CPAP therapy effectively improved the central airway narrowing in the saber-sheath trachea and air trapping in the peripheral airways, which were associated with acute bronchiolitis. Noninvasive respiratory management and treatment for airway infection have improved airway narrowing in this patient with saber-sheath trachea.
